# Disease Risks from Foods, England and Wales, 1996–2000

**DOI:** 10.3201/eid1103.040191

**Published:** 2005-03

**Authors:** Goutam K. Adak, Sallyanne M. Meakins, Hopi Yip, Benjamin A. Lopman, Sarah J. O'Brien

**Affiliations:** * Health Protection Agency Centre for Infections, London, United Kingdom

**Keywords:** Disease Risks from Foods, England and Wales, 1996–2000

Data from population-based studies and national surveillance systems were collated and analyzed to estimate the impact of disease and risks associated with eating different foods in England and Wales. From 1996 to 2000, an estimated 1,724,315 cases of indigenous foodborne disease per year resulted in 21,997 hospitalizations and 687 deaths. The greatest impact on the healthcare sector arose from foodborne *Campylobacter* infection (160,788 primary care visits and 15,918 hospitalizations), while salmonellosis caused the most deaths (209). The most important cause of indigenous foodborne disease was contaminated chicken (398,420 cases, risk [cases/million servings] = 111; case-fatality rate [deaths/100,000 cases] = 35, deaths = 141). Red meat (beef, lamb, and pork) contributed heavily to deaths, despite lower levels of risk (287,485 cases, risk = 24, case-fatality rate = 57, deaths = 164). Reducing the impact of indigenous foodborne disease is mainly dependent on controlling the contamination of chicken.

Foodborne infection is a major cause of illness and death worldwide (1–4). Recognizing this, the World Health Organization (WHO) developed its Global Strategy for Food Safety (1). In the developing world, foodborne infection leads to the death of many children (2), and the resulting diarrheal disease can have long-term effects on children's growth as well as on their physical and cognitive development (5,6). In the industrialized world, foodborne infection causes considerable illness, heavily affecting healthcare systems (3,4).

The WHO Global Strategy for Food Safety acknowledges, "Effective control of foodborne disease must be based on evaluated information about foodborne hazards and the incidence of foodborne disease." Estimates of the contributions of specific pathogens to the overall extent of foodborne infection at a national level are available (3,4). We refined the techniques used to estimate the acute health effects and the risks associated with consuming different foods. Our analyses should inform evidence-based control strategies for foodborne infection.

## Methods

### Indigenous Foodborne Disease

Indigenous foodborne disease is defined as food-related infectious gastroenteritis acquired and occurring in England and Wales. We derived pathogen-specific estimates for indigenous foodborne disease (Table 1) by using the method of Adak et al. (4) for the following 5 disease parameters: all disease, case-patients seen at a primary care setting (by general practitioners), hospitalizations, hospital occupancy, and deaths (Appendix, stages A–C).

**Table 1:** Estimated annual impact of indigenous foodborne disease by etiologic agent, England and Wales

Pathogen	Cases	General practitioner cases	Hospital		
Cases	Days	Deaths
Bacteria					
*Aeromonas* spp.	0	0	0	0	0
*Bacillus* spp.	10,717	4,287	26	67	0
*Campylobacter* spp.	337,655	160,788	15,918	58,897	80
*Clostridium perfringens*	168,436	88,651	709	10,496	177
*C. difficile* cytotoxin	0	0	0	0	0
*Escherichia coli* O157:H7	1,026	1,026	389	2,216	23
Non–O157:H7 STEC*	114	114	43	246	3
Other *E. coli*	62,050	13,850	319	1561	6
*Listeria monocytogenes*	221	221	221	3,959	78
Nontyphoidal salmonellae	73,193	52,280	2,666	15,465	209
*Salmonella* Typhi	86	86	35	239	0
*S*. Paratyphi	91	91	29	181	0
*Shigella* spp.	308	308	7	37	0
*Staphylococcus aureus*	9,196	3,678	232	278	0
*Vibrio cholerae* O1 and O139	0	0	0	0	0
*V. cholerae*, other serotypes	194	97	8	30	0
Other vibrio species	291	146	4	16	2
*Yersinia* spp.	129,338	11,054	619	5,448	3
Parasites					
*Cryptosporidium parvum*	1,699	894	32	119	3
*Cyclospora cayatenensis*	1,026	540	3	10	0
*Giardia lamblia*	1,999	1,052	6	22	0
Viruses					
Adenovirus 40/41	0	0	0	0	0
Astrovirus	17,741	4,032	12	47	4
Norovirus	61,584	9,775	39	152	10
Rotavirus	8,205	1,368	42	110	4
Sapovirus	0	0	0	0	0
Unknown	839,144	106,221	637	1,785	85
Total†	1,724,315	460,560	21,997	101,382	687

*STEC, Shiga toxin–producing *Escherichia coli*. †Totals are calculated on the basis of rounding to whole numbers.

### Foods Causing Indigenous Foodborne Disease

Outbreaks reported as foodborne, involving a single vehicle of infection and identified by epidemiologic or microbiologic investigations (N = 766, Table A1), were extracted from the National Surveillance Database for General Outbreaks of Infectious Intestinal Disease (GSURV) (7). Reported outbreaks in which investigators implicated either no (n = 612) or >1 (n = 234) vehicle of infection were excluded from these analyses. We also excluded outbreaks in which no pathogen was confirmed by laboratory testing (n = 113), although most of these outbreaks were suspected to be due to norovirus and were also linked to the same range of vehicles of infection. Foods were classified into broad food groups, such as poultry, and more specific food types, e.g., chicken (Table 2). A "complex foods" group was created to accommodate dishes consisting of ingredients of various food types in which the precise source of infection was not verified.

**Table 2:** Estimated annual impact of indigenous foodborne disease, by food group and type, England and Wales

Food group/type	Cases (%)	Deaths (%)	Case-fatality rate*
Poultry	502,634 (29)	191 (28)	38
Chicken	398,420 (23)	141 (21)	35
Turkey	87,798 (5)	45 (7)	52
Mixed/unspecified	16,416 (1)	4 (1)	27
Eggs	103,740 (6)	46 (7)	44
Red meat	287,485 (17)	164 (24)	57
Beef	115,929 (7)	67 (10)	58
Pork	46,539 (3)	24 (4)	53
Bacon/ham	17,450 (1)	9 (1)	53
Lamb	46,239 (3)	27 (4)	59
Mixed/unspecified	61,329 (4)	36 (5)	59
Seafood	116,603 (7)	30 (4)	26
Fish	22,311 (1)	10 (2)	47
Shellfish	77,019 (4)	16 (2)	21
Mixed/unspecified	17,273 (1)	4 (1)	24
Milk	108,043 (6)	37 (5)	34
Other dairy products	8,794 (0)	5 (0)	55
Vegetable/fruit	49,642 (3)	14 (2)	29
Salad vegetables	37,496 (2)	11 (2)	28
Cooked vegetables	6,870 (0)	2 (0)	35
Fruit	5,275 (0)	1 (0)	25
Rice	26,981 (2)	5 (1)	20
Complex foods	453,237 (26)	181 (26)	40
Infected food handler	67,157 (4)	14 (2)	20
Total†	1,724,315	687	40

*Deaths/100,000 cases. †Totals given are calculated on the basis of rounding to whole numbers.

We calculated the percentage of outbreaks due to each food type for each pathogen. For disease of unknown origin, we used the percentages as determined above for disease due to all known pathogens. These percentages were applied to the pathogen-specific estimates for the mean values for all disease, visits to general practitioners, hospitalizations, hospital occupancy, and deaths for the years 1996–2000 to produce pathogen-specific totals by food type for each of the 5 disease parameters used to describe the annual disease impact (Tables 2 and 3, Appendix, stage D). We then calculated food-specific totals for all disease, visits to general practitioners, hospitalizations, hospital occupancy, and deaths by adding together the appropriate food-specific totals for each pathogen (Appendix, stage E).

**Table 3:** Estimated annual healthcare impact of indigenous foodborne disease, by food group and type, England and Wales

Food group/type	General practitionercases (%)	Hospital cases (%)	Hospital days (%)
Poultry	159,433 (35)	9,952 (45)	41,645 (41)
Chicken	129,271 (28)	9,005 (41)	36,425 (36)
Turkey	23,679 (5)	360 (2)	3,001 (3)
Mixed/unspecified	6,483 (1)	587 (3)	2,219 (2)
Eggs	19,554 (4)	552 (3)	3,410 (3)
Red meat	80,805 (18)	1,231 (6)	10,935 (11)
Beef	34,981 (8)	429 (2)	4,284 (4)
Pork	11,923 (3)	219 (1)	1,685 (2)
Bacon/ham	4,470 (0)	82 (0)	632 (0)
Lamb	14,283 (3)	157 (1)	1,721 (2)
Mixed/unspecified	15,148 (3)	343 (2)	2,613 (3)
Seafood	23,998 (5)	828 (4)	3,690 (4)
Fish	4,603 (1)	112 (1)	748 (1)
Shellfish	12,861 (3)	134 (1)	752 (1)
Mixed/unspecified	6,534 (1)	582 (3)	2,190 (2)
Milk	40,755 (9)	3,681 (17)	14,176 (14)
Other dairy products	1,561 (0)	67 (0)	402 (0)
Vegetable/fruit	11,912 (3)	702 (3)	2,932 (3)
Salad vegetables	9,874 (2)	660 (3)	2,671 (3)
Cooked vegetables	1,184 (0)	27 (0)	168 (0)
Fruit	853 (0)	15 (0)	93 (0)
Rice	5,127 (1)	73 (0)	432 (0)
Complex foods	103,409 (22)	4,175 (19)	20,646 (20)
Infected food handler	14,007 (3)	736 (3)	3,113 (3)
Total*	460,560	21,997	101,382

*Totals given are calculated on the basis of rounding to whole numbers.

### Food-Specific Risk

The U.K. Government National Food Survey (8) collects population-based food consumption data. These data were used to calculate the number of servings of each food type consumed per resident for the period 1996–2000. These denominators were used to calculate food-specific risks, expressed as cases per million servings for all disease and hospitalizations per billion servings (Table 4, Appendix, stage F).

**Table 4:** Estimated risks associated with food groups and types, England and Wales

Food group/type	Disease risk*	Risk ratio	Hospitalization risk†	Risk ratio
Poultry	104	947	2,063	4,584
Chicken	111	1,013	2,518	5,595
Turkey	157	1,429	645	1,433
Mixed/unspecified	24	217	852	1,893
Eggs	49	448	262	583
Red meat	24	217	102	227
Beef	41	375	153	339
Pork	20	180	93	208
Bacon/ham	8	75	39	86
Lamb	38	343	128	285
Mixed/unspecified	17	157	96	214
Seafood	41	374	293	650
Fish	8	75	41	92
Shellfish	646	5,869	1,121	2,490
Mixed/unspecified	NA‡	NA	NA	NA
Milk	4	35	133	295
Other dairy products	2	17	14	32
Vegetable/fruit	1	NA	8	NA
Salad vegetables	6	53	103	229
Cooked vegetables	0	1	0	1
Fruit	0	2	1	1
Rice	11	101	30	67

*Cases/1 million servings. †Hospitalizations/1 billion servings. ‡NA, not applicable.

## Quality of Evidence

Each of the above steps was classified according to whether the pathogen-specific data elements used were direct measures, extrapolations, or inferences (Table 5). This classification system permitted us to evaluate the effects of potential biases on the final estimates produced.

**Table 5:** Quality of evidence

Stage	Data sources	Evidence	Principal assumptions	Potential effects of bias on final estimates
All infectious intestinal disease	Population studies	Measured	Representivity of data	Moderate
Etiology	Population studies	Measured for most; inferred rarely	Accuracy and sensitivity of diagnostic methods	Moderate
Indigenous infection	National laboratory report surveillance; special studies	Measured	Completeness of reporting	Negligible
Foodborne transmission	National outbreak surveillance (GSURV)*	Measured for most; inferred rarely	Representivity of data	Major
Food attribution	GSURV	Measured	Representivity of data	Major
Presentations to primary care	Population studies	Measured	Representivity of data	Moderate
Hospitalizations	GSURV; special studies	Measured	Representivity of data	Moderate
Hospital occupancy	Hospital episode statistics	Measured	Representivity of data	Moderate
Deaths	GSURV	Measured	Representivity of data	Negligible
Food specific risks	National food survey	Measured	Representivity of data	Major

*GSURV, National Surveillance Database for General Outbreaks of Infectious Intestinal Disease.

## Results

### Causes of Disease

Unknown agents accounted for 49% of all cases but only 23% of all visits to general practitioners, 3% of all hospitalizations, 2% of hospital occupancy, and 12% of all deaths (Table 1). *Campylobacter* spp. had the greatest effect on healthcare provision, according to all of the parameters examined. Nontyphoidal salmonellae and *Clostridium perfringens* caused most deaths. *Listeria monocytogenes* and *Escherichia coli* O157:H7 together accounted for 15% of all deaths but <0.1% of all cases.

### Disease Impact According to Food

Of the 1,724,315 estimated cases of indigenous foodborne disease in England and Wales, 67,157 (4%) were cases in which humans were considered to be the source of infection (foods contaminated by infected food handlers; Tables 2 and 3). Subtracting these cases left 1,657,158 cases in which contaminated food was the likely source. Within this subset, most illness was attributed to eating poultry (502,634, 30%), complex foods (453,237, 27%), and red meat (287,485, 17%). Only 76,623 (5%) patients were infected by eating plant-based foods, i.e., vegetables, fruit, and rice.

Chicken consumption accounted for more disease, deaths, and healthcare usage than any other food type. Milk also exerted a considerable impact on healthcare provision. No other single food type accounted for >8% for any of the healthcare use measures. In general, the healthcare impact arising from plant-based foods was low.

The lowest case-fatality rates were associated with plant-based foods. By contrast, foods of bovine origin tended to have the highest case-fatality rates. Shellfish had the lowest case-fatality rate of all of the foods of animal origin.

### Illness and Risk

Analysis by food group (Table 4) shows that vegetables and fruit had the lowest disease and hospitalization risks and poultry had the highest. Red meat accounted for more illness than seafood but was associated with a lower risk for disease (24 cases/million servings compared with 41 cases/million servings).

The lowest disease risk for a single food type was for cooked vegetables, at 0.11 cases/million servings. This risk was used to calculate disease risk ratios for the other food types. Disease risk ratios ranged from 2 for fruit to 5,869 for shellfish. Within individual food groups, large variations in disease risk ratios occurred. A disease risk ratio was not calculated for the vegetable and fruit food group because cooked vegetables contribute to the overall risk for the group.

The lowest hospitalization risk for a single food type was for cooked vegetables, 0.45 hospitalizations/billion servings. This risk was used to calculate hospitalization risk ratios for the other food types. While salad vegetables had a disease risk ratio of 53, the hospitalization risk ratio was 229. Chicken had the highest hospitalization risk ratio, 5,595. This figure is >4 times the value estimated for turkey and more than double the estimate for shellfish, both of which had higher disease risk ratios than chicken.

## Discussion

To our knowledge, our study is the first to examine the impact of and risk for indigenous foodborne disease by food type. When all parameters were considered, infection due to chicken was consistently responsible for more disease, while disease linked to plant-based foods had a minor impact on the population.

Our methods build on approaches to estimate the impact of foodborne diseases in the United States (3) and England and Wales (4). To minimize bias, we avoided using assumptions whenever possible. We concluded that the effects of bias on the etiologic data (Table 1) were moderate (Table 5) because we were able to estimate the incidence of disease for each agent by taking national laboratory surveillance data and applying pathogen-specific multiplication factors that had been determined through a large population-based study (9). We were also able to use direct measurements from special studies and national surveillance systems to estimate the impact of foreign travel. We avoided using expert opinion (Table 5). Techniques such as Delphi (10) are available to assimilate the judgments of expert panels to produce consensus data. However, the Delphi estimate for the incidence of salmonellosis due to the consumption of products made from chicken and eggs (10) in the United Kingdom was >3 times the incidence for all salmonellosis calculated from a national population-based incidence study (9).

The use of data from published outbreak investigations also presents difficulties. Comparing outbreak surveillance data with those from published reports demonstrates a bias that favors the publication of novel findings and exceptional events (11). Therefore, we only used contemporary data drawn from locally based surveillance systems, population-based studies, and surveys (Table 5) (4) in these analyses. Nevertheless, certain reservations apply when using outbreak surveillance data to estimate the proportion of disease due to each food type for each pathogen. Ideally, a full account should be taken of the relative pathogen-specific contributions of each food type to both sporadic and outbreak-associated disease. However, determining the proportion of cases that fall into these 2 categories for any pathogen is problematic.

For sound epidemiologic reasons, case-control studies of sporadic disease test specific hypotheses that might explain disease transmission (12–15). Sample sizes are determined to detect associations for major risk factors. Population-attributable fractions are calculable for only a small number of foods for the small number of pathogens studied with these methods. Each study delivers a snapshot of the epidemiology of disease at a point in time for a particular population. While some of the findings from these studies are generalizable, population-attributable fractions for individual foods are not because food production patterns and consumer preferences change from country to country and with time (8,16,17). Corroborative evidence to support identified associations between disease and food consumption for studies of sporadic disease is usually lacking. However, in outbreak investigations, microbiologic findings, production records, and the like lend weight to the inferences drawn from analytic epidemiology (18–20). We believe that the true impact of outbreak-associated disease has likely been greatly underestimated (21,22).

Accounting for disease caused by intermittent or unpredictable food processing failures is important. For example, an estimated 224,000 people throughout the United States were infected with *Salmonella enterica* serotype Enteritidis after eating ice cream that had become contaminated as a result of a processing failure (20). However, outbreak cases were only formally recognized in Minnesota. The scale of the outbreak emerged because of an unusually detailed epidemiologic investigation. Therefore, under normal circumstances, most of those affected would have been classified as sporadic cases. This outbreak alone would have accounted for 17% of the 1.3 million cases of foodborne salmonellosis in the United States for 1994 (3). The 1996/7 FoodNet case-control study did not find an association between pasteurized ice cream and sporadic salmonellosis (12) because the study was not conducted during the narrow timeframe when the implicated product was on the market. This example is not isolated; milk-processing failures have resulted in hundreds of outbreak cases of *Campylobacter* and *E. coli* O157:H7 infections in the United Kingdom (18). While outbreaks of this type continue to be identified through routine surveillance, others likely go undetected. However, testing for associations between apparently sporadic disease and consumption of contaminated "pasteurized" milk using case-control studies is difficult for several reasons: study participants are unaware of the process history of the milk that they drink; pasteurized milk is very commonly drunk and identifying differences in exposure rates would involve extremely large sample sizes; and since the geographic and temporal distribution of cases would be expected to be heterogeneous, studies would have to extend over long periods and large areas. For these reasons, recent case-control studies of sporadic *Campylobacter* and *E. coli* O157:H7 infections in the United Kingdom failed to show associations between disease and consumption of milk (13,14,23). Similar arguments apply for the role of fruit juice or sprouts in the transmission of *E. coli* O157:H7 (24,25) or salad vegetables and *Salmonella* serotypes (26). While all of these foods have made considerable, if intermittent, contributions to the overall impact of disease in the population, their role in sporadic disease is hard to test and has seldom been demonstrated. Thus, published case-control studies of sporadic infection provide insufficient applicable data for our purposes.

By contrast, GSURV is large, comprehensive, and provides contemporary locally defined evidence-based data that takes into account the contribution of a much broader range of foods. For example, the foods most frequently associated with disease in published studies of sporadic *Campylobacter* infection (15,23), i.e., chicken, pork, red meat, and unpasteurized milk, also feature most prominently in GSURV, but GSURV also takes into account the more minor contributions of foods such as salad vegetables, fruit, and seafood. However, for certain pathogens the amount of outbreak data available is limited. The food distribution percentages for *Campylobacter* were based on 28 outbreaks (Table A1). Therefore, we have exercised considerable caution in interpreting these data and have identified this area as one in which the effects of bias on the final estimates are likely to be most profound (Table 5). Nevertheless, the results are also plausible. In our analyses, chicken emerges as the most important contributor to *Campylobacter* infection. This finding is consistent with data from food and veterinary studies (27,28), evaluations of the interventions enforced after the Belgian dioxin crisis (29), and observations on the relationships between human infection and poultry operations in Iceland (30). Our estimates for impact and risk for disease linked to shell eggs is consistent with a U.S. Department of Agriculture risk assessment on *Salmonella* Enteritidis in shell eggs and egg products (31). Therefore, after taking all of these factors into account, we concluded that GSURV was the most suitable source of pathogen-specific risk exposure data.

Our analyses were based on data drawn from 766 outbreaks in which a single vehicle of infection was identified. The 612 outbreaks that were reported as foodborne but had no identified vehicle of infection were excluded from analysis. In effect, we have made the tacit assumption that distribution of foods in the subset of outbreaks in which a vehicle was identified is representative of the complete population of outbreaks. However, certain vehicles may be more likely to be implicated in outbreak investigations than others. This situation might occur if investigators tend to preferentially collect data on the types of food that are perceived as high risk or when laboratory methods vary in sensitivity according to food type. Therefore, a systematic vehicle detection bias could potentially result in our analyses underestimating the contribution and risks attributable to those foods that were rarely implicated in outbreak investigations, e.g., salad items such as sprouts, which are now being recognized as potential sources of infection (25), fruit, or background ingredients such as herbs and spices.

Eggs are used as an ingredient in a wide range of foods such as desserts, sauces, and savories (complex foods). These dishes always include other ingredients so ascribing disease-causing ingredients in the complex foods category is difficult. There are inherent difficulties in demonstrating epidemiologic association beyond the level of vehicle of infection to that of source. However, several factors (being seen by a general practitioner, hospitalization, and case-fatality rates) linked to complex foods are similar to those for eggs. Also, ≈70% of the complex foods associated with illness included eggs as an ingredient. Therefore, we suggest that eggs are probably a major source of infection for disease related to complex foods.

Eating shellfish was associated with the highest disease risk. Shellfish tends to be a luxury food, and consumption levels were low when compared with those of other food types. Although the number of cases attributed to shellfish was of the same order as beef or eggs, the level of risk was much higher. Preharvesting contamination of oysters with norovirus had a major impact in generating cases of disease. This finding presents an additional impact to that arising from the cross-contamination with *Salmonella* of ready-to-eat items such as cocktail shrimp (32).

When severity of illness data are taken into consideration, an elevated risk is associated with eating chicken. Chicken has a lower disease risk ratio than either shellfish or turkey but has a higher hospitalization risk ratio. This finding is explained by the relative prominence of *Campylobacter* and nontyphoidal salmonellae in illness attributable to chicken. Infection with these pathogens is much more likely to result in hospitalization than disease due to norovirus, which accounts for much shellfish-associated illness, or *C. perfringens*, one of the more common turkey-associated infections.

Risks associated with eating vegetables were generally low. However, risks associated with cooked vegetables were much lower than those associated with salad vegetables. This finding is mainly because cooking would normally eliminate the pathogens that can contaminate vegetables in the field, the processing plant, the market, or the kitchen through cross-contamination. However, no parallel control process exists for salad vegetables, which are generally regarded as ready to eat.

While these analyses provide data on the impact of disease attributable to different food types, considerable heterogeneity exists in the origin, production, and handling of each of these types of food. Further research is needed to examine the influence of imported foods, organic production, factory farming, and commercial catering.

We have also attempted to define the contribution of foods by infected food handlers. One of the key reasons for conducting these analyses was to provide an evidence base for developing disease control strategies. Controlling transmission of infection from infected food handlers in commercial and domestic catering requires different strategies than controlling foodborne zoonoses through the food chain. The pathogen most frequently transmitted by infected food handlers was norovirus. Given the ubiquity of norovirus infection (9,33), its extreme infectivity, and the sudden and violent onset of symptoms (34), control of transmission is difficult and more focused strategies are needed.

Our evidence-based analyses demonstrate that the most important priority in reducing the impact of indigenous foodborne disease in England and Wales is controlling infection from contaminated chicken. Chicken was associated with relatively high levels of risk and accounted for more disease, health service usage, and death than any other individual food type. Interventions introduced during the mid-1990s to control *S*. Enteritidis in the Great Britain chicken flock (35) appear to have been successful in reducing the burden of salmonellosis in England and Wales (4). These findings are consistent with analyses from Sweden (36), Denmark (37), and the United States (38), which together demonstrate that foodborne salmonellosis can be substantially reduced by implementing targeted initiatives to control *Salmonella* in domestic livestock.

The greatest challenge to protect the population from foodborne infection is to develop effective programs to control *Campylobacter* through the chicken production chain. This intervention is possible, as witnessed in Iceland, where measures at retail level and in the household were introduced to prevent *Campylobacter* transmission. Parallel declines (>70%) were subsequently observed in the carriage of *Campylobacter* in broiler flocks and in human infections (29). Finally, the data from Europe and the United States show that the largest benefits in reducing *Salmonella* and *Campylobacter* levels have come from implementing controls in farm-to-retail processing rather than in instituting them in domestic kitchens, where the estimated impacts are much smaller in scale (39), although still important.

## Appendix

### Methods Formulas

(Example: impact of disease attributable to the consumption of chicken, pathogen 1 = nontyphoidal salmonellae)**A) All infectious intestinal disease (IDD) due to pathogen 1, 1996–2000**C*_P1_* = L*_P1_* x a*_P1_*(All IID due to nontyphoidal salmonellae 1996–2000, 464,549 = 119,115 x 3.9)Where: C*_P1_* = cases of disease due to pathogen 1L*_P1_* = laboratory reports for pathogen 1A*_P1_* = ascertainment ratio for pathogen 1**B) Indigenous infectious intestinal disease due to pathogen 1, 1996–2000**C_P1,E_ = C_P1_ x e_P1_(All indigenous IID due to nontyphoidal salmonellae 1996–2000, 399,480 = 464,549 x 85.99%)Where: C*_P1_*_,_*_E_* = cases of indigenously acquired disease due to pathogen 1e*_P1_* = percentage of disease due to pathogen 1 acquired in England and Wales**C) Indigenous foodborne disease (IFD) due to pathogen 1, 1996–2000**C_P1,E,F_ = C_P1,E_ x f_P1_(All IFD due to nontyphoidal salmonellae 1996–2000, 365,963 = 399,480 x 91.61%)Where: C_P1,E,F_ = cases of IFD due to pathogen 1f_P1_ = percentage of foodborne transmission for disease due to pathogen 1**Ci)** G_P1,E,F_ = C_P1,E,F_ x g_P1_(IFD cases due to nontyphoidal salmonellae seen by general practitioners, 1996–2000, 261,402 = 365,963 x 71.42%)Where: G_P1,E,F_ = general practitioner cases of IFD due to pathogen 1g_P1_ = percentage of cases that present to general practice for pathogen 1**Cii)** H_P1,E,F_ = G_P1,E,F_ x h_P1_(IFD hospitalizations due to nontyphoidal salmonellae, 1996–2000, 13,332 = 261,402 x 5.10%)Where: H_P1,E,F_ = hospitalized cases of IFD due to pathogen 1h_P1_ = percentage of GP cases that are hospitalized for pathogen 1**Ciii)** B_P1,E,F_ = H_P1,E,F_ x b_P1_(IFD hospital occupancy due to nontyphoidal salmonellae 1996–2000, 77,323 = 13,332 x 5.8)Where: B_P1,E,F_ = Bed days in hospital for IFD due to pathogen 1b_P1_ = average hospital stay for illness due to pathogen 1**Civ)** D_P1,E,F_ = G_P1,E,F_ x d_P1_(Deaths from IFD due to nontyphoidal salmonellae, 1996–2000, 1,046 = 261,402 x 0.40%)Where: D_P1,E,F_ = Deaths from IFD due to pathogen 1d_P1_ = percentage deaths in general practitioner cases for pathogen 1D) IFD per year due to pathogen 1 attributable to food 1*C_AnnP1,E,FI_ = (C_P1,E,F_ x fI_P1_)/5(All IFD per year due to nontyphoidal salmonellae attributable to chicken, 13,169 = [365,963 x 17.99%]/5)Where: C*_AnnP1_*_,_*_E_*_,_*_FI_* = cases of IFD per year due to pathogen 1 attributable to food 1fI*_P1_* = percentage of IFD due to pathogen 1 attributable to food 1E) All cases of IFD per year attributable to food 1*C*_AnnE,FI_* = C*_AnnP1_*_,_*_E_*_,_*_FI_* + C*_AnnP2_*_,_*_E_*_,_*_FI_*  + C*_AnnP3_*_,_*_E_*_,_*_FI_* C*_AnnPn,E,FI_*(All cases of IFD per year attributable to chicken, 398,420 = 13,169_salmonella_ + 168,828*_Campylobacter_*_sp_ + 25,478 *_Cl. perfringens_* + 3,824_otherbacteria_ + 10,224_viruses_ + 176,897_unknown_)Where: C*_AnnE_*_,_*_FI_* = all cases per year of IFD attributable to food 1**F) Risk of IFD associated with the consumption of food 1**R_Ann,FI_ = C_AnnE,FI_/S_E,FI_(Risk of IFD attributed to chicken 111.39 = 398,420/3,576.87)Where: R_AnnE,FI_ = risk for IFD associated with the consumption of food 1(expressed as cases per million servings)S_E,FI_ = total servings of food 1 (expressed by the million)1 serving = 140 g for poultry, red meat and fish/shellfish, 200 mL for milk, 150 g for dairy produce, 63 g for eggs, 80 g for vegetables, fruit, and rice*Analagous calculations were used to produce estimates for general practitioner case-patients, hospital case-patients/occupancy, deaths, and hospitalization risk.

## Appendix

**Table A1:** General outbreaks of infectious iIntestinal disease iInvolving 1 food vehicle, England and Wales

Food group	All salmonellae (%)*	*Campylobacter* (%)	Other bacteria (%)	Viruses (%)	Protozoa (%)
Poultry	108 (23)	15 (54)	49 (25)	4 (6)	0 (0)
Red meat	51 (11)	0 (0)	83 (42)	0 (0)	0 (0)
Eggs	69 (14)	0 (0)	0 (0)	0 (0)	0 (0)
Seafood	19 (4)	1 (4)	2 (1)	23 (36)	0 (0)
Milk	8 (2)	6 (21)	9 (5)	0 (0)	1 (100)
Other dairy products	4 (1)	0 (0)	2 (1)	0 (0)	0 (0)
Vegetables/fruit	10 (2)	1 (4)	5 (3)	6 (9)	0 (0)
Rice	4 (2)	0 (0)	12 (6)	0 (0)	0 (0)
Complex foods	202 (42)	4 (14)	32 (16)	11 (17)	0 (0)
Infected food handler	3 (1)	1 (4)	1 (1)	20 (31)	0 (0)
Total	478	28	195	64	1

*Percentages are rounded to the nearest whole number.
